# Cannabidiol prevents methamphetamine-induced neurotoxicity by modulating dopamine receptor D1-mediated calcium-dependent phosphorylation of methyl-CpG-binding protein 2

**DOI:** 10.3389/fphar.2022.972828

**Published:** 2022-09-06

**Authors:** Baoyu Shen, Ruilin Zhang, Genmeng Yang, Yanxia Peng, Qianyun Nie, Hao Yu, Wenjuan Dong, Bingzheng Chen, Chunhui Song, Yan Tian, Lixiang Qin, Junjie Shu, Shijun Hong, Lihua Li

**Affiliations:** Key Laboratory of Drug Addiction Medicine of National Health Commission (NHC), School of Forensic Medicine, Kunming Medical University, Kunming, China

**Keywords:** methamphetamine, cannabidiol, dopamine receptor D1, methyl-CpG-binding protein 2, calcium

## Abstract

In the past decade, methamphetamine (METH) abuse has sharply increased in the United States, East Asia, and Southeast Asia. METH abuse not only leads to serious drug dependence, but also produces irreversible neurotoxicity. Currently, there are no approved pharmacotherapies for the treatment of METH use disorders. Cannabidiol (CBD), a major non-psychoactive (and non-addictive) cannabinoid from the cannabis plant, shows neuroprotective, antioxidative, and anti-inflammatory properties under METH exposure. At present, however, the mechanisms underlying these properties remain unclear, which continues to hinder research on its therapeutic potential. In the current study, computational simulations showed that CBD and METH may directly bind to the dopamine receptor D1 (DRD1) via two overlapping binding sites. Moreover, CBD may compete with METH for the PHE-313 binding site. We also found that METH robustly induced apoptosis with activation of the caspase-8/caspase-3 cascade *in-vitro* and *in-vivo*, while CBD pretreatment prevented these changes. Furthermore, METH increased the expression of DRD1, phosphorylation of Methyl-CpG-binding protein 2 (MeCP2) at serine 421 (Ser421), and level of intracellular Ca^2+^ *in-vitro* and *in-vivo*, but these effects were blocked by CBD pretreatment. The DRD1 antagonist SCH23390 significantly prevented METH-induced apoptosis, MeCP2 phosphorylation, and Ca^2+^ overload *in-vitro*. In contrast, the DRD1 agonist SKF81297 markedly increased apoptosis, MeCP2 phosphorylation, and Ca^2+^ overload, which were blocked by CBD pretreatment *in-vitro*. These results indicate that CBD prevents METH-induced neurotoxicity by modulating DRD1-mediated phosphorylation of MeCP2 and Ca^2+^ signaling. This study suggests that CBD pretreatment may resist the effects of METH on DRD1 by competitive binding.

## Introduction

Methamphetamine (METH) use has markedly increased in North America, East Asia, and Southeast Asia in recent years (UNODC, 2021). Of concern, the continuous use of METH can trigger serious neurotoxicity (Kim et al., 2020; Jayanthi et al., 2021), ultimately leading to neurological impairment, tissue damage, and neuropsychological disturbance (Cadet and Krasnova, 2009; Lappin et al., 2018; Shukla and Vincent, 2020). Evidence suggests that METH-induced neurotoxicity involves many mechanisms (Jayanthi et al., 2021), including dopamine (DA) release, oxidative stress, mitochondrial stress, endoplasmic reticulum (ER) stress, microglial and astrocyte activation, ubiquitin/proteasome system (UPS) dysfunction, immediate early gene (IEG) modification, and autophagy. However, the specific mechanisms underlying METH-induced neurotoxicity remain poorly understood.

As a methyl DNA-binding transcriptional regulator, methyl-CpG-binding protein 2 (MeCP2) participates in transcription repression (Jones et al., 1998; Nan et al., 1998) and activation (Chahrour et al., 2008), RNA splicing regulation (Young et al., 2005), and transcriptional noise suppression (Skene et al., 2010). MeCP2 is also a critical regulator of neurodevelopment and adult brain function (Gulmez Karaca et al., 2019), and dysfunction in MeCP2 can cause Rett syndrome and other neuropsychiatric disorders (Amir et al., 1999; Chin and Goh, 2019). Furthermore, MeCP2-mediated neurotoxicity may also contribute to neuropsychiatric disorders (Russell et al., 2007; Dastidar et al., 2012; Montgomery et al., 2018) and participate in METH-induced behavioral disorders in rodents (Lewis et al., 2016; Wu et al., 2016; Fan et al., 2020). However, the role of MeCP2-mediated neurotoxicity in METH use disorders remains poorly understood. Phosphorylation has been suggested as a potential mechanism by which MeCP2 modulates gene expression (Chao and Zoghbi, 2009; Damen and Heumann, 2013). The phosphorylation of MeCP2 (pMeCP2) at serine 80 (Ser80) increases MeCP2 binding to target gene promoters and restricts transcription, while phosphorylation of MeCP2 at serine 421 (Ser421) increases MeCP2 dissociation from promoters and transcription activation (Chen et al., 2003; Chao and Zoghbi, 2009). Phosphorylation of MeCP2 at Ser421 is mediated by a calcium-dependent mechanism (Chen et al., 2003; Buchthal et al., 2012). Phosphorylation of MeCP2 at Ser421 contributes to neural and behavioral responses to psychostimulants in mice, whereas phosphorylation of MeCP2 in the nucleus accumbens (NAc) is mediated by D1-like DA receptors, including DA receptors D1 (DRD1) and D5 (DRD5) (Deng et al., 2010). There is growing evidence that both calcium signaling and DRD1 are involved in METH-induced neurotoxicity (Ares-Santos et al., 2012; Ares-Santos et al., 2013; Friend and Keefe, 2013; Andres et al., 2015; Sun et al., 2015; Nguyen et al., 2018), and activation of DRD1 can significantly induce neuronal damage (Park et al., 2019). Therefore, DRD1-mediated phosphorylation of MeCP2 may be a critical mechanism in METH-induced neurotoxicity.

Cannabidiol (CBD) is a primary non-psychoactive cannabinoid in the cannabis plant and exhibits considerable therapeutic potential in the treatment of neuropsychiatric disorders, including epilepsy, Parkinson’s disease (PD), Alzheimer’s disease (AD), depression, anxiety, psychosis, and drug dependence (Elsaid et al., 2019; Premoli et al., 2019; Vitale et al., 2021). Recent research has indicated that CBD exerts neuroprotective effects on METH-induced neurotoxicity in rats (Razavi et al., 2021). Furthermore, some evidence has suggested that CBD modulates several receptors in METH exposure, such as D1-like DA receptors (Nouri et al., 2021; Shen et al., 2022), D2-like DA receptors (Hassanlou et al., 2021), Sigma-1 receptors (Yang et al., 2020), and Toll-like type-4 receptors (TLR4) (Majdi et al., 2019). We previously showed that CBD may modulate DRD1 to attenuate METH-induced DA release (Shen et al., 2022). However, the underlying mechanism related to the neuroprotective effects of CBD on METH-induced neurotoxicity remains elusive. In the current study, we hypothesized that METH induces neurotoxicity *via* DRD1-mediated phosphorylation of MeCP2 at Ser421 with calcium influx, and CBD treatment prevents METH-induced neurotoxicity *via* modulation of DRD1.

## Materials and methods

### Molecular docking

Molecular docking was performed using AutoDock v4.2.6 in accordance with previous research (Bian et al., 2019; Zhang et al., 2021). Structural information on rat DRD1 was obtained from the AlphaFold Protein Structure Database (https://alphafold.ebi.ac.uk/), and structural information on METH and CBD was obtained from PubChem (https://pubchem.ncbi.nlm.nih.gov/). First, preparation of DRD1 was performed using AutoDockTools v1.5.6, including the addition of hydrogens and calculation of atomic charges with the Kollman all-atom approach (Bian et al., 2019). The atomic charges of CBD and METH were calculated using the Gasteiger-Hückel approach with AutoDockTools v1.5.6 (Bian et al., 2019). A three-dimensional (3D) search grid with 40 × 44 × 40 points was created using the AutoGrid algorithm, and the maximum number of poses per ligand was set to 200. Next, all postures for CBD and METH were docked using AutoDock v4.2.6. The docking parameters were set to default, as per previous research (Zhang et al., 2021). The docking scores of all postures were extracted using AutoDockTools v1.5.6. The affinity score was greater than −5 kcal/mol, indicating a reliable docking process. The best docking posture was then selected. Finally, binding site analysis was performed using Discovery Studio Visualizer v4.5 and visualized using PyMol v2.5.2.

### Drugs

For the *in-vitro* experiments, methamphetamine hydrochloride (National Institutes for Food and Drug Control, #171212–200603, Beijing, China) was dissolved in phosphate-buffered saline (PBS; pH 7.2; Biological Industries, #02-024-1ACS, Beit HaEmek, Israel). SCH23390 hydrochloride (Bio-Techne, #0925, Minneapolis, MN, United States) or SKF81297 hydrobromide (Bio-Techne, #1447, Minneapolis, MN, United States) was dissolved in PBS with gentle warming. In addition, CBD (Pulis Biological Technology Co. Ltd., #PD0155, Chengdu, China) was dissolved in dimethyl sulfoxide (DMSO) (MP Biomedicals, #196055, CA, United States). The final concentration of DMSO in the culture medium was not more than 0.1%. For the *in-vivo* experiments, methamphetamine hydrochloride was dissolved in saline and CBD was mixed with 5% DMSO and 5% Tween-80 (Solarbio Life Sciences, #T8360, Beijing, China) in saline with gentle warming. Preparation of CBD was based on research from other laboratories (Hampson et al., 1998; Ryan et al., 2009; Hay et al., 2018; Luján et al., 2018) and our previous studies (Yang et al., 2020; Shen et al., 2022).

### Primary culture of neurons

Neurons for primary culture were isolated from the brain of early postnatal Sprague-Dawley rats following previous research, with slight modification (Chen et al., 2016; Li et al., 2019; Ding et al., 2020). Briefly, mesaticephalic and cortical tissues were gathered from brains of early postnatal rats and digested with trypsin solution (Gibco, #25200–072, Grand Island, NE, United States of America). Tissue mixtures were filtered with a 70-μm cell strainer (Biosharp Life Sciences, #BS-70-XBS, Hefei, China) and centrifuged at 3,000 *× rpm* for 5 min at 4°C. The sediments were resuspended in high glucose culture medium (Biological Industries, #06-1055-57-1ACS, Beit HaEmek, Israel) with 10% fetal bovine serum (Biological Industries, #04-001-1ACS, Beit HaEmek, Israel) and 2% penicillin/streptomycin (Gibco, #15140-122, Grand Island, NE, United States). After 24-h *in-vitro* culture, the medium was replaced with neurobasal medium (STEMCELL Technologies, #05790, Canada) containing 10% fetal bovine serum, 2% B-27 (Gibco, #17504-044, Grand Island, NE, United States), 1% glutamine (Gibco, #35050-061, Grand Island, NE, United States), and 2% penicillin/streptomycin, which was replaced every 3 days. We identified the purity of primary neurons before all experiments in our previous work (Shen et al., 2022). Therefore, the results of neuronal purity are reported in our earlier manuscript (Shen et al., 2022). For the cell viability test, neurons were treated with 50, 100, 200, 400, 800, or 1,000 μM METH for 24 h, and subsequently treated with 400 μM METH for 3, 6, 12, 24, or 48 h. In addition, 0.1, 1, or 10 μM CBD was used as a pretreatment for 1 h before the addition of METH. For the DRD1 experiment, 10 μM SCH23390 (DRD1 antagonist) was used as a pretreatment for 1 h before the addition of METH, and 10 μM SKF81297 (DRD1 agonist) was used for 1 h after the addition of CBD. The concentrations were based on research from other laboratories, with slight modification (Sun et al., 2015; Chakraborty et al., 2016; Chen et al., 2016; Uno et al., 2017; Branca et al., 2019).

### Cell counting kit-8 assay

Neurons were seeded at a density of 5 × 10^3^ cells/well in a 96-well plate. After the neurons were incubated with drugs following the above procedures, cell viability was detected using Cell counting kit-8 (CCK-8) reagent (Dalian Meilun Biotechnology Co. Ltd., #MA0218, Dalian, China). Absorbance was obtained on a microplate reader at 450 nm. Cell viability was calculated according to the formula provided by the kit.

### Western blotting

Neurons and brain tissue from rats were suspended in RIPA lysis buffer (Beyotime, #P0013B, Shanghai, China) containing protease inhibitors (Beyotime, #ST506-2, 1:100, Shanghai, China) and phosphatase inhibitor cocktail A (Beyotime, #P1082, 1:50, Shanghai, China). After the neurons and tissue samples were homogenized using a sonicator, the homogenate was lysed for 30 min on ice. The lysates were then centrifuged at 12,000 *× g* for 15 min at 4°C, and the supernatant was subjected to BCA protein assay (Beyotime, #P0012, Shanghai, China) to determine protein concentration. Total protein (25 μg) was separated by 12% sodium dodecyl sulfate-polyacrylamide gel electrophoresis (SDS-PAGE) (Beyotime, #P0012AC, Shanghai, China) and transferred onto polyvinylidene fluoride (PVDF) membranes (Sigma-Aldrich, IPVH00010, St. Louis, MO, United States). The membranes were blocked with 5% dry skimmed milk in Tris-buffered saline and incubated overnight at 4°C in blocking solution containing primary antibodies against DRD1 (Bio-Techne, #NB110-60017, 1:800, Minneapolis, MN, United States), pMeCP2 (phospho Ser421) (Abcam, #ab254050, 1:1,000, Cambridge, United Kingdom), caspase-8 (Abcam, #ab25901, 1:1,000, Cambridge, United Kingdom), cleaved caspase-8 (Proteintech, #66093-1-Ig, 1:2,000, IL, United States), caspase-3 (Cell Signaling Technology, #9662, 1:1,000, MA, United States), cleaved caspase-3 (Cell Signaling Technology, #9664, 1:1,000, MA, United States), and β-actin (Proteintech, #66009–1, 1:5,000, IL, United States). After rinsing three times with Tris-buffered saline containing Tween-20 (TBST) (Solarbio Life Sciences, #T8220, Beijing, China), the membranes were incubated with rabbit horseradish peroxidase (HRP) (Cell Signaling Technology, #7074S, 1:5,000, MA, United States) or mouse HRP secondary antibodies (Cell Signaling Technology, #7076S, 1:5,000, MA, United States), followed by rinsing three times with TBST. Immunoreactive proteins were detected using a chemiluminescence reaction (Biosharp Life Sciences, #BL520A, Hefei, China). Images were obtained using an immunoblotting detection system (Bio-Rad Laboratories, Hercules, CA, United States) and analyzed with ImageJ software.

### Immunofluorescence staining

After incubation with drugs, the neurons were fixed in 4% paraformaldehyde (Biosharp Life Sciences, #BL539A, Hefei, China) for 30 min and rinsed three times with PBS. For immunolabeling, the neurons were first penetrated with 0.2% Triton X-100 (Solarbio Life Sciences, #T8200, Beijing, China) for 30 min, then blocked with 10% goat serum (Solarbio Life Sciences, #SL038, Beijing, China) for 2 h at room temperature. The neurons were then incubated with PBS containing antibodies against DRD1 (Bio-Techne, #NB110-60017, 1:200, Minneapolis, MN, United States), pMeCP2 (phospho Ser421) (Abcam, #ab254050, 1:200, Cambridge, United Kingdom), cleaved caspase-8 (Proteintech, #66093-1-Ig, 1:200, IL, United States), or cleaved caspase-3 (Cell Signaling Technology, #9664, 1:200, MA, United States) overnight at 4°C. Correspondingly, species-specific fluorescent-conjugated secondary antibodies were applied to detect immunoreactive proteins. The nucleus was labeled with DAPI (Cell Signaling Technology, #8961S, MA, United States). Images were obtained using a fluorescent microscope (Olympus Corporation, #BX53, Tokyo, Japan) and analyzed with ImageJ.

### Intracellular Ca^2+^ detection

Intracellular Ca^2+^ was detected as per previous study (Sun et al., 2015). For the *in-vitro* experiments, drug-treated neurons were rinsed three times with Hanks’ balanced salt solution (HBSS, Sigma-Aldrich, #H6648, St. Louis, MO, United States) and incubated with HBSS containing 10 μM Fluo-3-AM solution (Sigma-Aldrich, #39294, St. Louis, MO, United States) for 60 min at 37°C. For the *in-vivo* experiments for Ca^2+^ detection, tissue sections were first penetrated with HBSS containing 0.2% Triton X-100 and 0.1% sodium citrate (Solarbio Life Sciences, #C1032, Beijing, China) for 30 min, then incubated with HBSS containing 10 μM Fluo-3-AM solution for 60 min at 37°C. After incubation with Fluo-3-AM solution, the neurons and tissue sections were rinsed three times with HBSS. Observations were performed with a fluorescent microscope at a detection wavelength of 488 nm. Obtained images were analyzed with ImageJ.

### Terminal deoxynucleotidyl transferase dUTP nick-end labeling staining

Apoptosis was detected using a Terminal Deoxynucleotidyl Transferase dUTP Nick-end Labeling (TUNEL) assay kit (Sigma-Aldrich, #11684817 910 or #12156792910, St. Louis, MO, United States) according to the manufacturer’s protocols. For the *in-vitro* experiments, neurons were fixed in 4% paraformaldehyde for 30 min and rinsed with PBS. For the *in-vivo* experiments, tissue sections were rinsed three times with PBS. After incubation with 3% H_2_O_2_ for 10 min at room temperature, the neurons and tissue sections were rinsed with PBS, then penetrated with PBS containing 0.2% Triton X-100 and 0.1% sodium citrate for 2 min on ice. For TUNEL staining, the samples were incubated with TUNEL reaction mixture for 60 min at 37°C. After the samples were rinsed three times with PBS, DAPI was used to localize nuclei. A fluorescent microscope was used to obtain images with the detection wavelength at 488 nm or 580 nm. DAPI- and TUNEL-positive cells were counted using ImageJ (*n* = 5). TUNEL-positive cell ratio = TUNEL-positive cells/DAPI-positive cells.

### Animals

Adult male Sprague-Dawley rats (8–9 weeks old, *n* = 40) were purchased from the Laboratory Animal Center, Kunming Medical University. The rats were housed in a humidity- (50% ± 10%) and temperature-controlled (22°C ± 1°C) room on a 12-h reverse light/dark cycle. Food and water were available *ad libitum*. All animal procedures were conducted according to the National Institutes of Health (NIH) Guide for the Care and Use of Laboratory Animals and were approved by the Animal Care and Use Committee of Kunming Medical University (permit number: kmmu2020403). The rats were randomly distributed into four groups: i.e., Saline (*n* = 10), CBD 40 mg/kg (*n* = 10), METH 15 mg/kg (*n* = 10), and CBD 40 mg/kg + METH 15 mg/kg (*n* = 10) groups.

### Animal treatments

After 1 week of habituation, the rats received intraperitoneal injections of METH (eight injections, 15 mg/kg/injection, 12-h intervals). Injections were performed at 08:00 and 20:00 for 4 days. This exposure paradigm was selected based on previous studies (Qiao et al., 2014; Huang et al., 2015; Xie et al., 2018; Xu et al., 2018; Huang et al., 2019; Chen et al., 2020). CBD was injected intraperitoneally at a dose of 40 mg/kg 1 h before METH administration, following our previous studies (Yang et al., 2020; Shen et al., 2022). During treatment, body weights were recorded every day. In addition, METH-stereotyped behavior was observed as described previously (Sams-Dodd, 1998; Roberts et al., 2010; Qiao et al., 2014). Briefly, an observer blind to the experiment visually assessed several classified behaviors, including: (0) No repetitive head movements; 1) Weak repetitive side-to-side head movements; 2) Strong repetitive side-to-side head movements; and 3) Stationary stereotyped behavior with strong side-to-side or circular head movements. Rats were anesthetized 24 h after the last injection, and perfused transcardially with saline. After perfusion with saline, the brains of rats were removed, and the prefrontal cortex (PFC) and hippocampus (Hip) were dissected bilaterally on ice and stored at −80°C for detection of proteins. For tissue sections, the rats were perfused transcardially with 4% paraformaldehyde and brains were preserved in 4% paraformaldehyde at 4°C. After 2 weeks of fixation, the brains were sunk in increasing concentrations of sucrose (10%, 20%, and 30%). Brains were sectioned (15 μm) along the coronal plane incorporating the PFC and Hip on a freezing microtome (Leica Biosystems, #CM 1860, Nussloch, Germany) kept at −20°C.

### Statistical analyses

All data were analyzed using SPSS v26.0 and presented as mean ± standard deviation (SD). One-way analysis of variance (ANOVA) was used to analyze data from parts A and B of the CCK-8 assay, with factors defined as METH treatments (corresponding to “METH dose” and “treatment time” levels). Three-way repeated-measures ANOVA was used to analyze data from the behavioral experiment, with factors defined as CBD treatment, METH treatment, and Time. Two-way ANOVA (no repeated measures) was used to analyze data from other *in-vitro* and *in-vivo* experiments. For the first part of the *in-vitro* experiments and other *in-vivo* experiments, factors were defined as CBD and METH treatments. For the second part of the *in-vitro* experiments, factors were defined as SCH23390 and METH treatments. For the third part of the *in-vitro* experiments, factors were defined as CBD and SKF81297 treatments. ANOVA was followed by Tukey HSD *post-hoc* tests. Before ANOVA, normality and homogeneity of equal variance were confirmed. The level of significance was set to 0.05.

## Results

### Methamphetamine and cannabidiol bind with DA receptors D1

**FIGURE 1:**
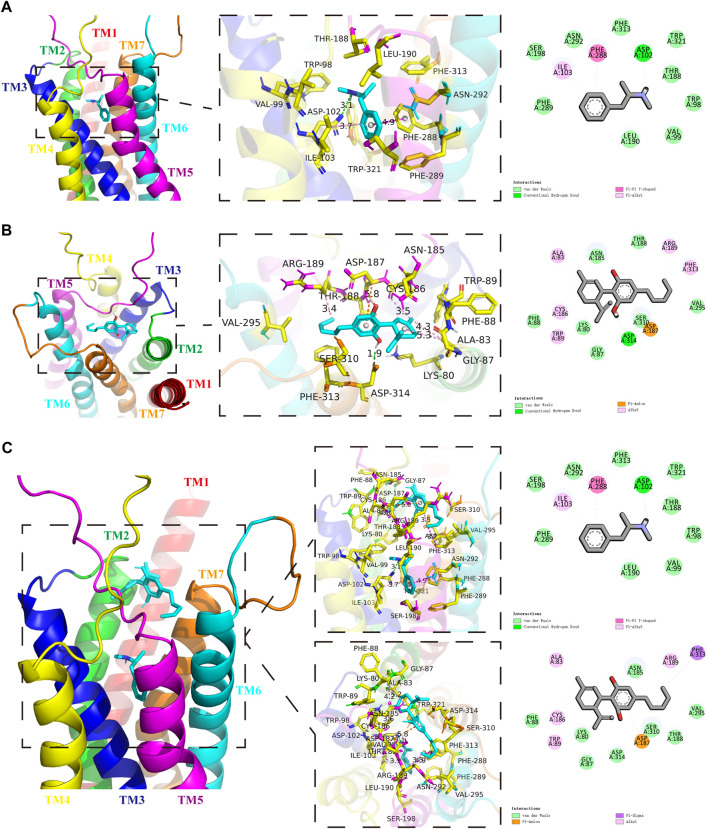
Protein-drug binding simulations. Seven transmembrane domains of DRD1 are shown as multicolor bands. METH or CBD are marked in cyan, while key DRD1 residues are marked in yellow. **(A)** Docking pose, binding sites, and interactions between DRD1 model and METH. **(B)** Docking pose, binding sites, and interactions between DRD1 model and CBD. **(C)** Docking pose, binding sites, and interactions among CBD, METH, and DRD1 model.

We first screened the METH-binding pocket in the DRD1 model from 7 reliable binding pockets. We chose the METH-binding pocket with the highest affinity (−5.8 kcal/mol). Specially, 12 amino acid residues of DRD1 formed the boundary of the pocket, including PHE-289, SER-198, ILE-103, ASN-292, PHE-288, PHE-313, ASP-102, TRP-321, THR-188, TRP-98, VAL-99, and LEU-190 (Figure 1A). Among these amino acids, one hydrogen bond was observed between residue ASP-102 from transmembrane (TM) 3 and METH at 3.1 Å. In addition, residue PHE-288 from TM6 and METH formed a Pi-Pi T-shaped non-covalent interaction at 4.9 Å, while residue ILE-103 from TM3 and METH formed a Pi-Alkyl non-covalent interaction at 3.7 Å (Figure 1A). We also performed docking simulation with the DRD1 model and CBD. In total, 15 reliable CBD-binding pockets were found, with the highest affinity of −7.3 kcal/mol. The best CBD-binding pocket in the DRD1 model consisted of 14 amino acid residues, including ALA-83, ASN-185, ARG-189, PHE-313, VAL-295, THR-188, SER-310, ASP-187, ASP-314, GLY-87, LYS-80, TRP-89, CYS-186, and PHE-88 (Figure 1B). A hydrogen bond was observed between residue ASP-314 from TM7 and CBD at 1.9 Å, while residue ASP-187 from TM5 and CBD formed a Pi-Anion non-covalent interaction at 5.8 Å. In addition, several Alkyl-Alkyl interactions were found between CBD and TRP-89, CYS-186, ALA-83, ARG-189, and PHE-313. To determine whether CBD competes with METH for binding sites, we docked METH and CBD with DRD1 based on the above pockets simultaneously. Results showed that CBD was bound to DRD1 near the METH-binding pocket (Figure 1C). Interestingly, for CBD and DRD1 interactions, the hydrogen bond between residue ASP-314 and CBD (Figure 1B) was converted to van der Waals force (Figure 1C), while the Alkyl-Alkyl interaction between residue PHE-313 (Figure 1B) was transformed into a Pi-Sigma covalent bond (Figure 1C). However, no differences were observed in the interactions between METH and DRD1 compared with METH docking alone (Figure 1C). Overlaps between CBD-binding sites and METH-binding sites were found, e.g., PHE-313 and THR-188 (Figure 1C). The bond strength between CBD and DRD1 was stronger than that of METH in PHE-313, but not that of METH in THR-188 (Figure 1C). This is likely because the covalent Pi-Sigma bond is stronger than van der Waals force for drug loading (Li and Yang, 2015).

### Cannabidiol prevented methamphetamine-induced decrease in cell viability

**FIGURE 2:**
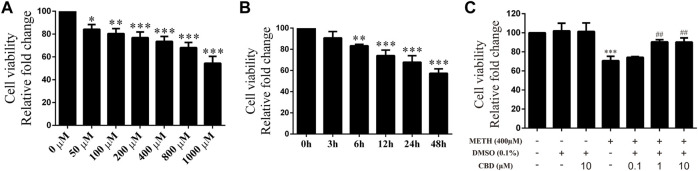
Cell viability of neurons incubated with different drugs. **(A)** Cell viability of neurons incubated with METH for 24 h at different concentrations (50, 100, 200, 400, 800, and 1,000 μM). **(B)** Cell viability of neurons incubated with 400 μM METH at different time points (3, 6, 12, 24, and 48 h). **(C)** Cell viability of neurons incubated with CBD (0.1, 1, and 10 μM) and/or METH (400 μM). ^*^ *p <* 0.05, ^**^ *p <* 0.01, or ^***^ *p <* 0.001 compared with control group, ^##^ *p <* 0.01 compared with METH group; Tukey HSD *post-hoc* comparison after significant ANOVA.

Cultured neurons were treated with various concentrations of METH (50, 100, 200, 400, 800, 1,000 μM) for 24 h, with cell viability then detected using the CCK-8 assay. Cell viability decreased gradually with the increase in METH concentration [Figure 2A; F (6, 14) = 30.603, *p <* 0.05, *p <* 0.01, *p <* 0.001]. Based on morphological changes in neurons and other previous studies (Sun et al., 2015; Chen et al., 2016), the 400 μM concentration of METH was chosen for subsequent experiments. The neurons were exposed to 400 μM METH for different times (3, 6, 12, 24, 48 h). As indicated in Figure 2B, cell viability showed a significant and time-dependent decrease [Figure 2B; F (5, 12) = 36.953, *p <* 0.01, *p <* 0.001]. These results suggest that METH induces neuronal death in a dose-dependent and time-dependent manner. As cellular damage was easy to detect, we also selected the 400 μM concentration of METH to incubate neurons for 24 h. Before METH exposure, the neurons were incubated with CBD (0.1, 1, and 10 μM) or vehicle (0.1% DMSO) for 1 h. Results showed that cell viability diminished significantly after 400 μM METH exposure [Figure 2C; F (6, 14) = 18.449, *p <* 0.001], increased markedly with CBD pretreatment (≥1 μm) [Figure 2C; F (6, 14) = 18.449, *p <* 0.01], and showed no change under CBD or vehicle alone [Figure 2C; F (6, 14) = 18.449, *p* = 1.000, *p* = 0.999]. Therefore, CBD appears to show a neuroprotective effect on METH exposure. Based on our findings and previous research (Branca et al., 2019), the 1 μM concentration of CBD was chosen to perform subsequent experiments.

### Cannabidiol blocked expression of DA receptors D1, phosphorylation of MeCP2, and apoptosis-related proteins induced by methamphetamine exposure *in-vitro*

**FIGURE 3:**
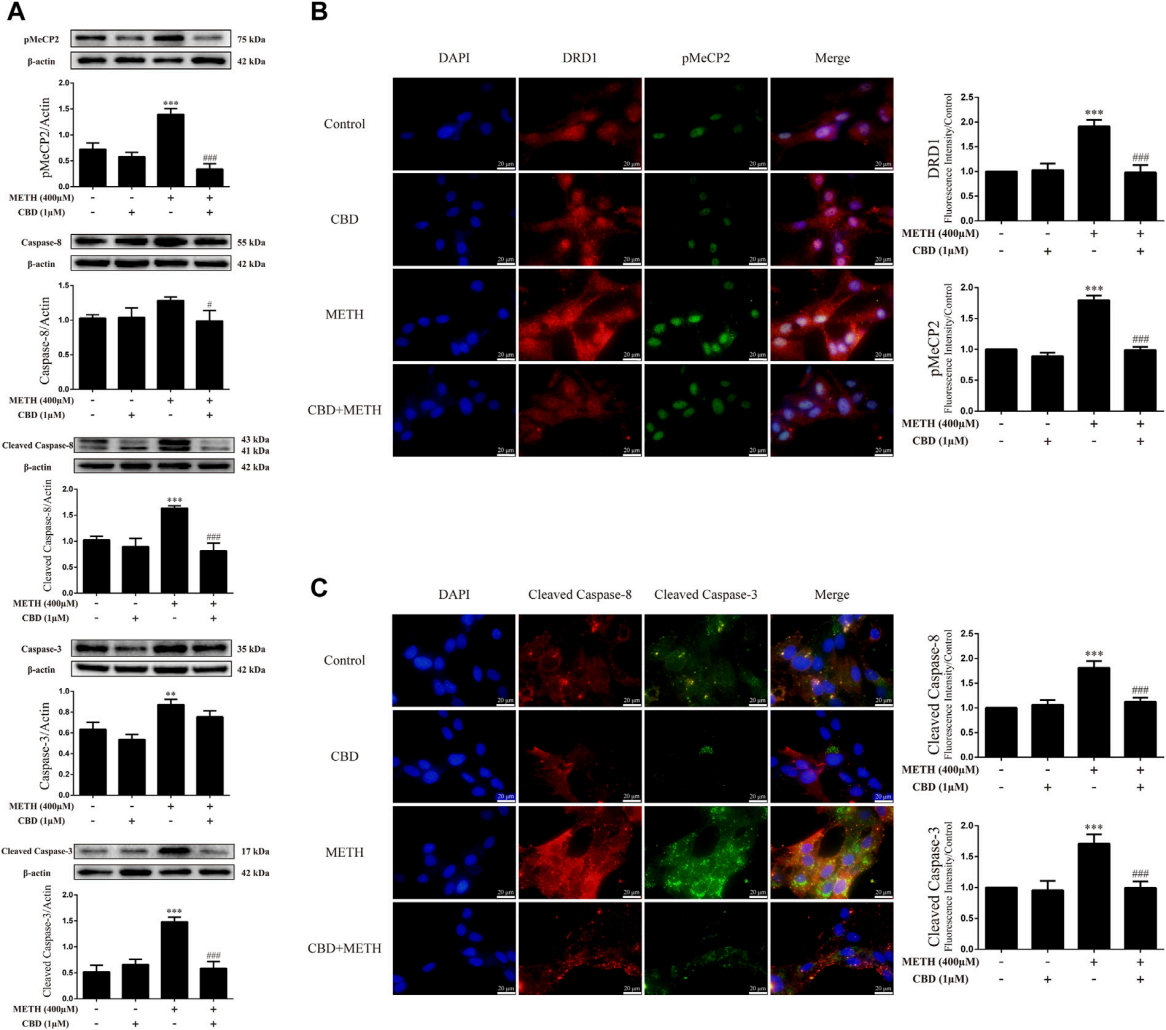
Protein levels and colocalization in neurons incubated with CBD and/or METH. **(A)** Levels of pMeCP2 and apoptosis-related proteins. **(B)** DRD1 and pMeCP2 levels and their colocalization. **(C)** Cleaved caspase-8 and cleaved caspase-3 levels and their colocalization. ^**^ *p <* 0.01 or ^***^ *p <* 0.001 compared with control group, ^#^ *p <* 0.05 or ^###^ *p <* 0.001 compared with METH group; Tukey HSD *post-hoc* comparison after significant ANOVA.

We next explored the mechanisms underlying the neuroprotective effects of CBD. To corroborate the cell viability findings, we detected the expression levels of apoptosis-related proteins (caspase-8, cleaved caspase-8, caspase-3, and cleaved caspase-3) in primary neurons. We found that METH significantly induced the expression levels of cleaved caspase-8 [Figure 3A; F (3, 8) = 30.841, *p <* 0.001], caspase-3 [Figure 3A; F (3, 8) = 20.211, *p <* 0.01], and cleaved caspase-3 [Figure 3A; F (3, 8) = 48.091, *p <* 0.001] *in-vitro*, but had no significant effect on caspase-8. However, CBD pretreatment prevented the high levels of cleaved caspase-8 [Figure 3A; F (3, 8) = 30.841, *p <* 0.001] and cleaved caspase-3 [Figure 3A; F (3, 8) = 48.091, *p <* 0.001] induced by METH. These findings were further verified by immunofluorescence staining [Figure 3C; F (3, 16) = 85.584, *p <* 0.001; F (3, 16) = 47.347, *p <* 0.001]. METH administration also significantly induced phosphorylation of MeCP2 at Ser421 [Figure 3A; F (3, 8) = 55.573, *p <* 0.001], which was prevented by CBD pretreatment [Figure 3A; F (3, 8) = 55.573, *p <* 0.001]. In addition, DRD1 and pMeCP2 were also expressed in the primary neurons (Figure 3B). Significant increases in DRD1 [Figure 3B; F (3, 16) = 71.533, *p <* 0.001] and pMeCP2 [Figure 3B; F (3, 16) = 320.272, *p <* 0.001] were observed after METH administration, but were prevented by CBD pretreatment [DRD1; Figure 3B; F (3, 16) = 71.533, *p <* 0.001; pMeCP2; Figure 3B; F (3, 16) = 320.272, *p <* 0.001]. These results suggest that the neuroprotective effects of CBD may involve DRD1 levels and MeCP2 phosphorylation at Ser421 in neurons.

### Cannabidiol prevented Ca^2+^ overload and apoptosis induced by methamphetamine in neurons

**FIGURE 4:**
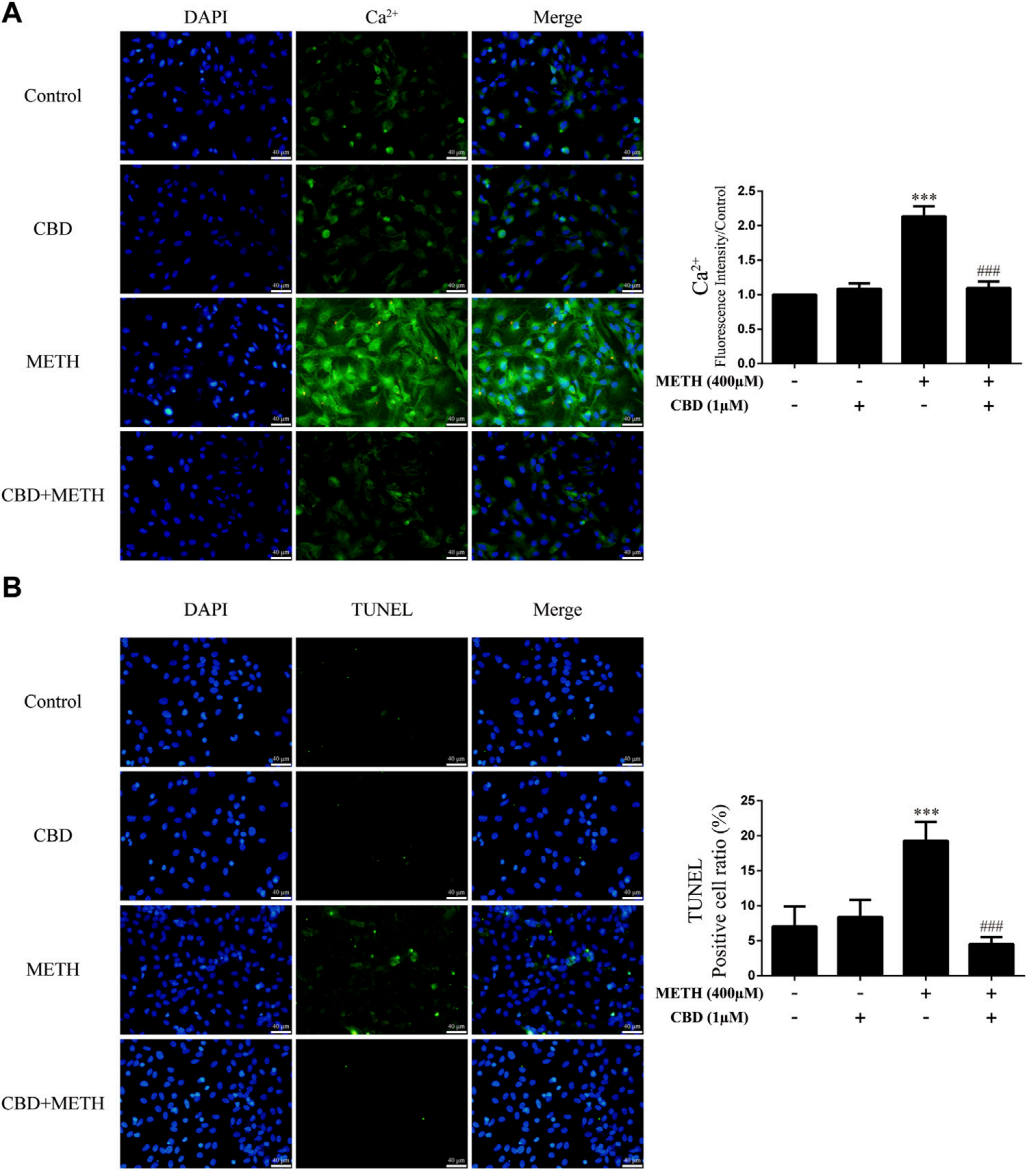
Intracellular Ca^2+^ and apoptosis levels in neurons incubated with CBD and/or METH. **(A)** Apoptosis level in neurons. **(B)** Intracellular Ca^2+^ level in neurons. ^***^ *p <* 0.001 compared with control group, ^###^ *p <* 0.001 compared with METH group; Tukey HSD *post-hoc* comparison after significant ANOVA.

As the phosphorylation of MeCP2 at Ser421 is calcium-dependent (Chen et al., 2003; Buchthal et al., 2012), we further investigated the level of intracellular Ca^2+^ in neurons. Results showed that METH significantly increased intracellular Ca^2+^ in the neurons [Figure 4A; F (3, 16) = 159.367, *p <* 0.001], whereas CBD pretreatment prevented the high level of Ca^2+^ [Figure 4A; F (3, 16) = 159.367, *p <* 0.001]. We applied TUNEL staining to detect neuronal apoptosis. Results showed a significant increase in the level of apoptosis after METH administration [Figure 4B; F (3, 16) = 37.384, *p <* 0.001], but a significant decrease in apoptosis with CBD pretreatment [Figure 4B; F (3, 16) = 37.384, *p <* 0.001]. Thus, intracellular Ca^2+^ may play an important role in the neuroprotective effects of CBD.

### Inhibition of DA receptors D1 activity prevented phosphorylation of MeCP2 and apoptosis-related protein expression induced by methamphetamine *in-vitro*

**FIGURE 5:**
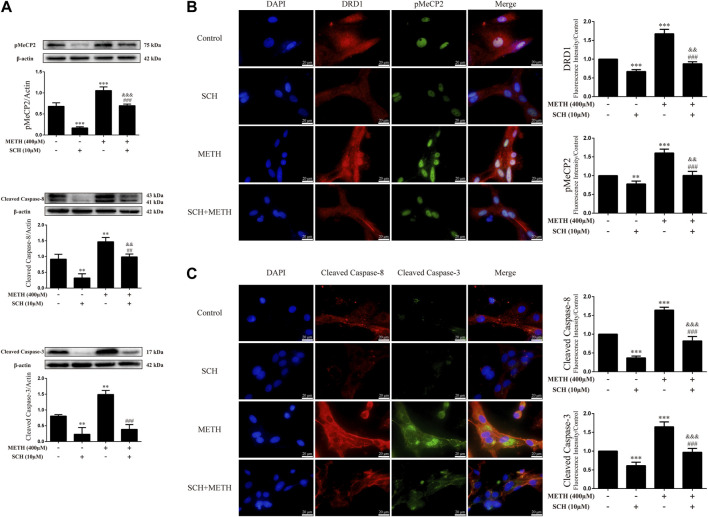
Protein levels and colocalization in neurons incubated with SCH23390 and/or METH. **(A)** Levels of pMeCP2 and apoptosis-related proteins. **(B)** DRD1 and pMeCP2 levels and their colocalization. **(C)** Cleaved caspase-8 and cleaved caspase-3 levels and their colocalization. ^**^ *p <* 0.01 or ^***^ *p <* 0.001 compared with control group, ^##^ *p <* 0.01 or ^###^ *p <* 0.001 compared with METH group, ^&&^ *p <* 0.01 or ^&&&^ *p <* 0.001 compared with SCH23390 group; Tukey HSD *post-hoc* comparison after significant ANOVA.

To clarify whether DRD1 signaling mediates apoptosis induced by METH, we applied the DRD1 antagonist SCH23390 to inhibit DRD1 activity in the neurons. Results showed that SCH23390 not only prevented METH-induced expression of cleaved caspase-8 [Figure 5A; F (3, 8) = 37.934, *p <* 0.01] and cleaved caspase-3 [Figure 5A; F (3, 8) = 44.214, *p <* 0.01, *p <* 0.001], but also blocked the phosphorylation of MeCP2 [Figure 5A; F (3, 12) = 127.199, *p <* 0.001] induced by METH. In addition, the phosphorylation of MeCP2 [Figure 5A; F (3, 12) = 127.199, *p* < 0.001] and levels of cleaved caspase-8 [Figure 5A; F (3, 8) = 37.934, *p* < 0.01] and cleaved caspase-3 [Figure 5A; F (3, 8) = 44.214, *p* < 0.01] were lower than that in the control when SCH23390 was administered alone. Immunofluorescence staining showed the preventive effects of SCH23390 on DRD1 expression [Figure 5B; F (3, 16) = 194.609, *p <* 0.001]. The pMeCP2 [Figure 5B; F (3, 16) = 89.432, *p <* 0.01, *p <* 0.001], cleaved caspase-8 [Figure 5C; F (3, 16) = 255.105, *p <* 0.001], and cleaved caspase-3 [Figure 5C; F (3, 16) = 103.056, *p <* 0.001] results were confirmed by immunofluorescence staining. Thus, METH appears to induce neuronal apoptosis *via* DRD1-mediated phosphorylation of MeCP2. However, DRD1 antagonist SCH23390 did not completely block the METH induction of DRD1 expression [Figure 5B; F (3, 16) = 194.609, *p <* 0.01], pMeCP2 expression [Figure 5A; F (3, 12) = 127.199, *p* < 0.001; Figure 5B; F (3, 16) = 89.432, *p <* 0.01], cleaved caspase-8 expression [Figure 5A; F (3, 8) = 37.934, *p <* 0.01; Figure 5C; F (3, 16) = 255.105, *p <* 0.001], or cleaved caspase-3 expression [Figure 5C; F (3, 16) = 103.056, *p <* 0.001]. Therefore, other pathways may also be involved in METH-induced caspase cascade.

### Repression of DA receptors D1 activity prevented Ca^2+^ overload and apoptosis induced by methamphetamine in neurons

**FIGURE 6:**
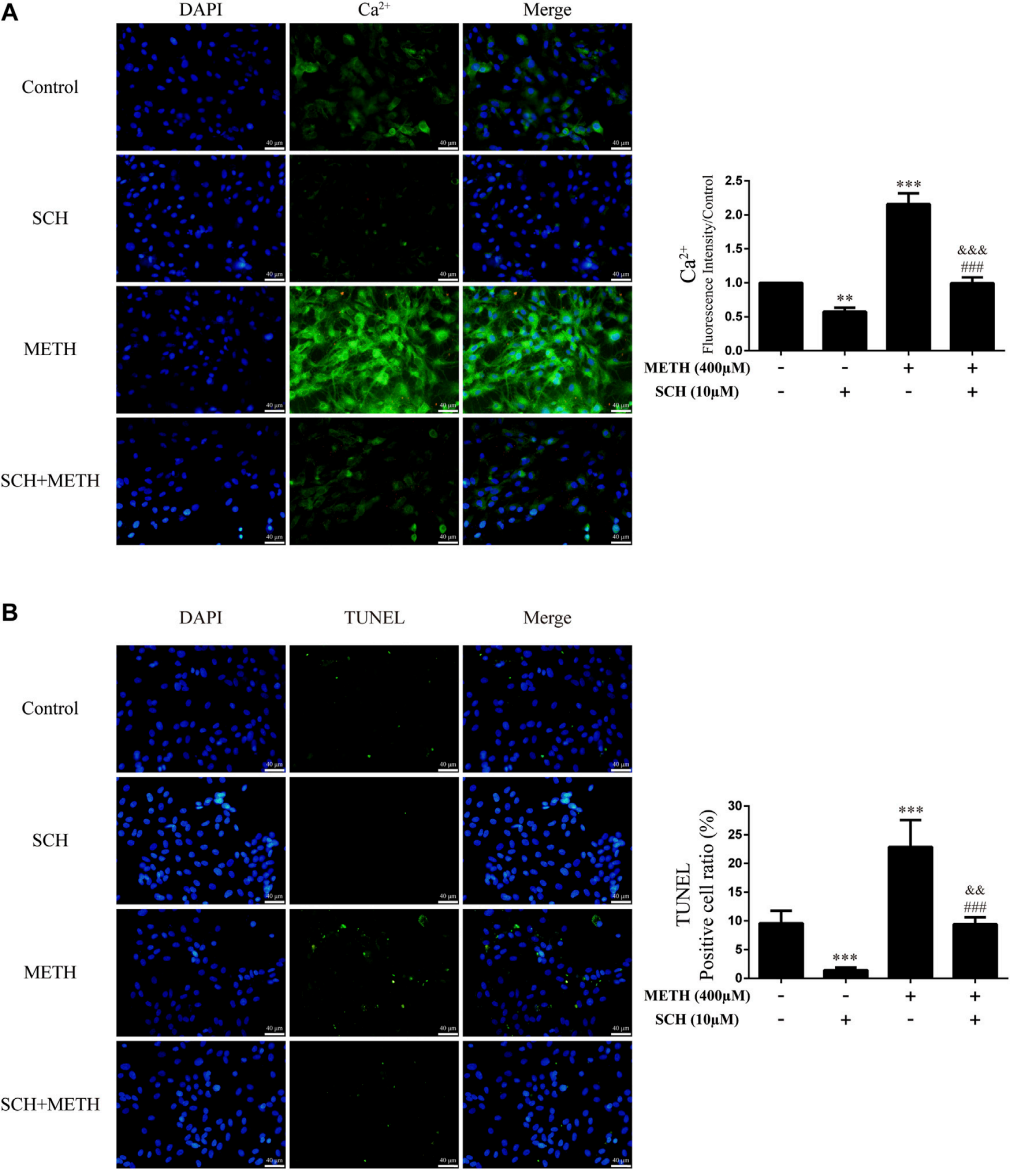
Intracellular Ca^2+^ and apoptosis levels in neurons incubated with SCH23390 and/or METH. **(A)** Apoptosis level in neurons. **(B)** Intracellular Ca^2+^ level in neurons. ^**^ *p <* 0.01 or ^***^ *p <* 0.001 compared with control group, ^###^ *p <* 0.001 compared with METH group, ^&&^ *p <* 0.01 or ^&&&^ *p <* 0.001 compared with SCH23390 group; Tukey HSD *post-hoc* comparison after significant ANOVA.

We next investigated intracellular Ca^2+^ and apoptosis levels in neurons when DRD1 activity was inhibited. We found that inhibition of DRD1 activity prevented METH-induced Ca^2+^ overload [Figure 6A; F (3, 16) = 256.570, *p <* 0.001] and inhibited METH-induced apoptosis [Figure 6B; F (3, 16) = 56.240, *p <* 0.001]. Furthermore, the neuronal levels of Ca^2+^ [Figure 6A; F (3, 16) = 256.570, *p <* 0.01] and apoptosis [Figure 6B; F (3, 16) = 56.240, *p <* 0.001] were also reduced compared with the control when administered SCH23390 alone. These results suggest that METH induces neuronal apoptosis *via* DRD1-mediated Ca^2+^ influx. Similarly, the levels of intracellular Ca^2+^ [Figure 6A; F (3, 16) = 256.570, *p <* 0.001] and apoptosis [Figure 6B; F (3, 16) = 56.240, *p <* 0.01] in the SCH23390 + METH group were higher than those in the SCH23390 group. Therefore, other pathways may also contribute to METH-induced Ca^2+^ influx and apoptosis.

### Cannabidiol prevented phosphorylation of MeCP2 and apoptosis-related protein expression induced by DA receptors D1 activation *in-vitro*

**FIGURE 7:**
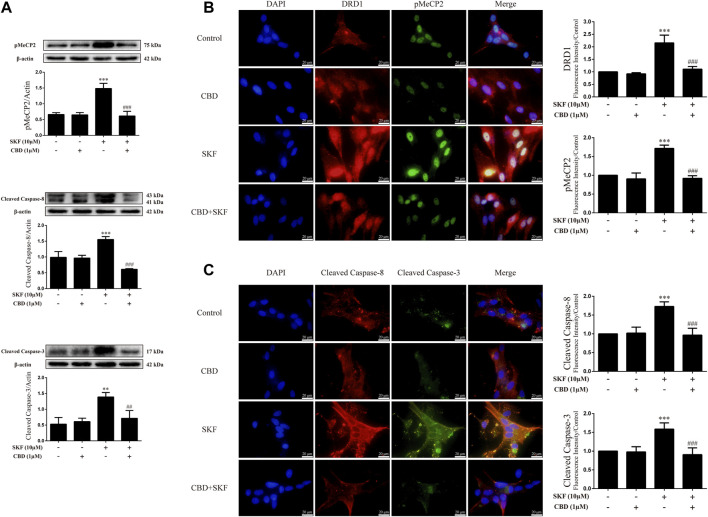
Protein levels and colocalization in neurons incubated with CBD and/or SKF81297. **(A)** Levels of pMeCP2 and apoptosis-related proteins. **(B)** DRD1 and pMeCP2 levels and their colocalization. **(C)** Cleaved caspase-8 and cleaved caspase-3 levels and their colocalization. ^**^ *p <* 0.01 or ^***^ *p <* 0.001 compared with control group, ^##^ *p <* 0.01 or ^###^ *p <* 0.001 compared with METH group; Tukey HSD *post-hoc* comparison after significant ANOVA.

To determine whether the neuroprotective effects of CBD work by inhibition of DRD1 signaling, we used the DRD1 agonist SKF81297 to activate DRD1. Results showed that DRD1 activation increased the expression levels of cleaved caspase-8 [Figure 7A; F (3, 12) = 48.763, *p <* 0.001] and cleaved caspase-3 [Figure 7A; F (3, 8) = 13.524, *p <* 0.01] and induced the phosphorylation of MeCP2 [Figure 7A; F (3, 8) = 37.637, *p <* 0.001]. As expected, CBD pretreatment prevented the high SKF81297-induced expression of pMeCP2 [Figure 7A; F (3, 8) = 37.637, *p <* 0.001], cleaved caspase-8 [Figure 7A; F (3, 12) = 48.763, *p <* 0.001], and cleaved caspase-3 [Figure 6A; F (3, 8) = 13.524, *p <* 0.01]. Immunofluorescence staining also showed that SKF81297 robustly stimulated DRD1 [Figure 7B; F (3, 16) = 60.311, *p <* 0.001], which was blocked by CBD pretreatment [Figure 7B; F (3, 16) = 60.311, *p <* 0.001]. MeCP2 phosphorylation also increased significantly [Figure 7B; F (3, 16) = 80.637, *p <* 0.001], but was prevented by CBD pretreatment [Figure 7B; F (3, 16) = 80.637, *p <* 0.001]. Cleaved caspase-8 [Figure 7C; F (3, 16) = 36.566, *p <* 0.001] and cleaved caspase-3 [Figure 7C; F (3, 16) = 24.377, *p <* 0.001] showed the same immunofluorescence staining results. Therefore, CBD appears to prevent neuronal apoptosis *via* DRD1-mediated phosphorylation of MeCP2.

### Cannabidiol blocked Ca^2+^ overload and apoptosis induced by DA receptors D1 activation in neurons

**FIGURE 8:**
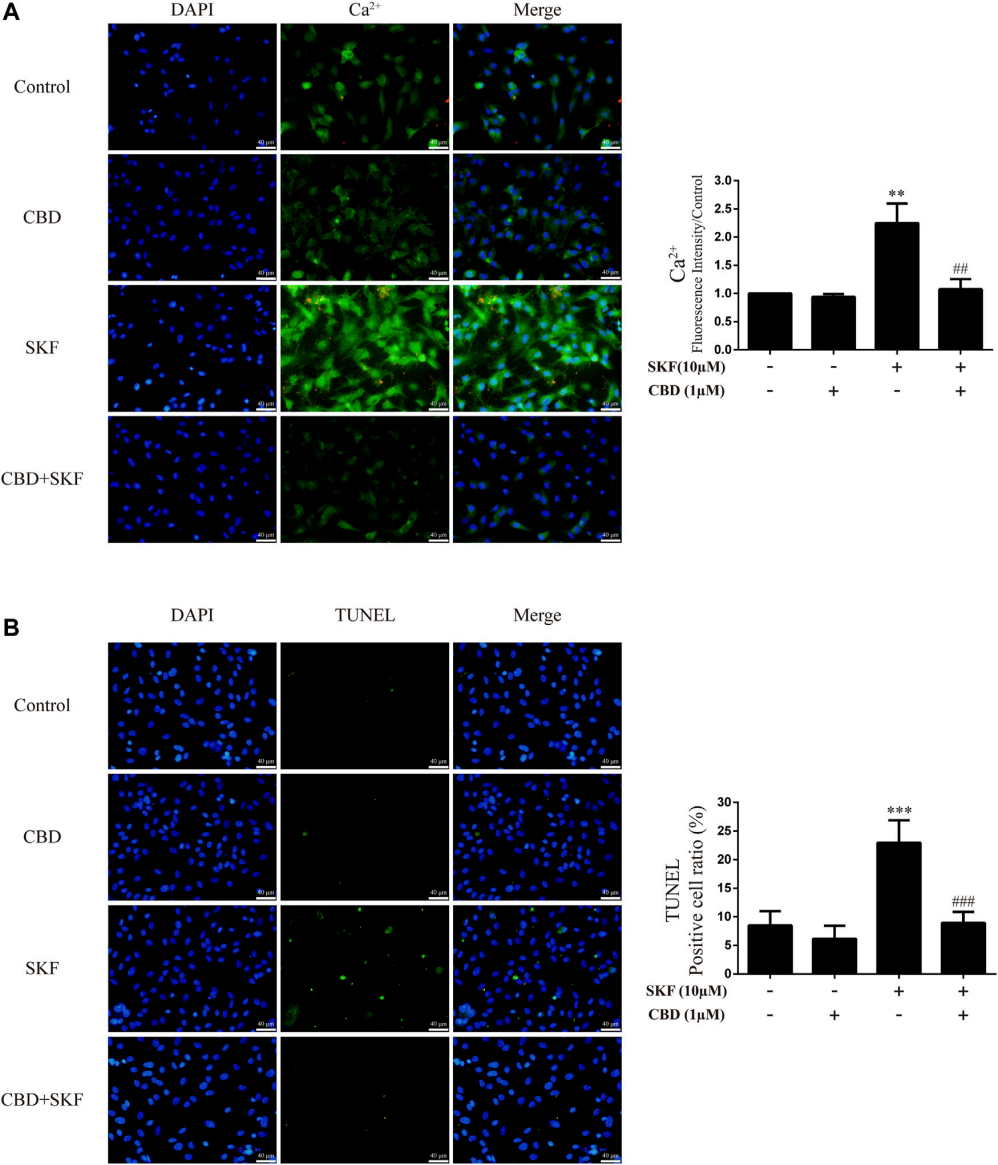
Intracellular Ca^2+^ and apoptosis levels in neurons incubated with CBD and/or SKF81297. **(A)** Apoptosis level in neurons. **(B)** Intracellular Ca^2+^ level in neurons. ^**^ *p <* 0.01 or ^***^ *p <* 0.001 compared with control group, ^##^ *p <* 0.01 or ^###^ *p <* 0.001 compared with METH group; Tukey HSD *post-hoc* comparison after significant ANOVA.

We further investigated the effects of DRD1 activation on intracellular Ca^2+^ and apoptosis in neurons. Results showed that DRD1 activation significantly induced neuronal Ca^2+^ influx [Figure 8A; F (3, 16) = 50.118, *p <* 0.01] and apoptosis [Figure 8B; F (3, 16) = 38.310, *p <* 0.001], but both were blocked by CBD pretreatment [Ca^2+^; Figure 8A; F (3, 16) = 50.118, *p <* 0.01]; apoptosis; [Figure 8B; F (3, 16) = 38.310, *p <* 0.001]. These results suggest that the neuroprotective effects of CBD work by preventing DRD1-mediated Ca^2+^ influx.

### Cannabidiol prevented growth inhibition and stereotyped behavior induced by methamphetamine in rats

**FIGURE 9:**
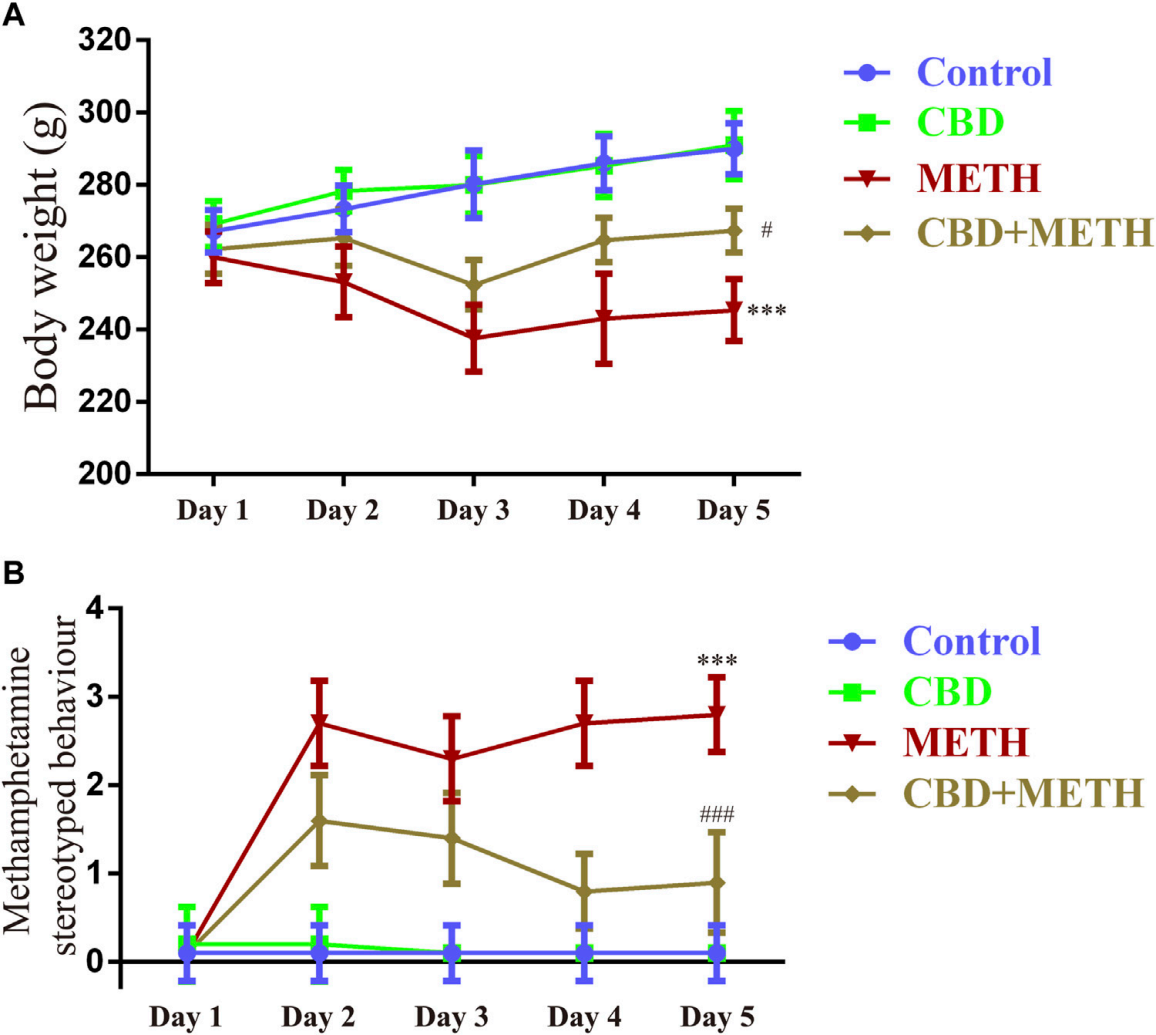
Changes in body weight and stereotyped behavior in rats repeatedly exposed to CBD and/or METH. **(A)** Changes in body weight in rats. **(B)** Changes in stereotyped behavior in rats. ^***^ *p <* 0.001 compared with control group, ^#^ *p <* 0.05 or ^###^ *p <* 0.001 compared with METH group; Tukey HSD *post-hoc* comparison after significant ANOVA.

To corroborate the findings that CBD resists neurotoxicity induced by METH *in-vitro*, we constructed a rat model using METH. We first investigated the effects of drug exposure on body weight in rats. Compared with the control group, repeated METH exposure inhibited normal weight gain in rats [Figure 9A; F (1, 16) = 25 383.728, *p <* 0.001], while multiple CBD pretreatments prevented the inhibition induced by METH [Figure 9A; F (1, 16) = 25 383.728, *p <* 0.05], although there was no significant effect when treated with CBD alone [Figure 9A; F (1, 16) = 25 383.728, *p* = 0.990]. We further investigated METH-induced stereotyped behavior during drug administration. Results showed that repeated METH exposure robustly induced stereotyped behavior in rats [Figure 9B; F (1, 36) = 279.914, *p <* 0.001] compared with the control group. However, CBD pretreatment blocked this METH-induced stereotyped behavior [Figure 9B; F (1, 36) = 279.914, *p <* 0.001], although CBD treatment alone had no effect [Figure 9B; F (1, 36) = 279.914, *p* = 0.992]. These results indicate that CBD exerts a global interventional effect on METH use disorders, including individual growth and behavioral changes.

### Cannabidiol prevented DA receptors D1 expression, phosphorylation of MeCP2, Ca^2+^ overload, and apoptosis induced by methamphetamine in prefrontal cortex of rats

**FIGURE 10:**
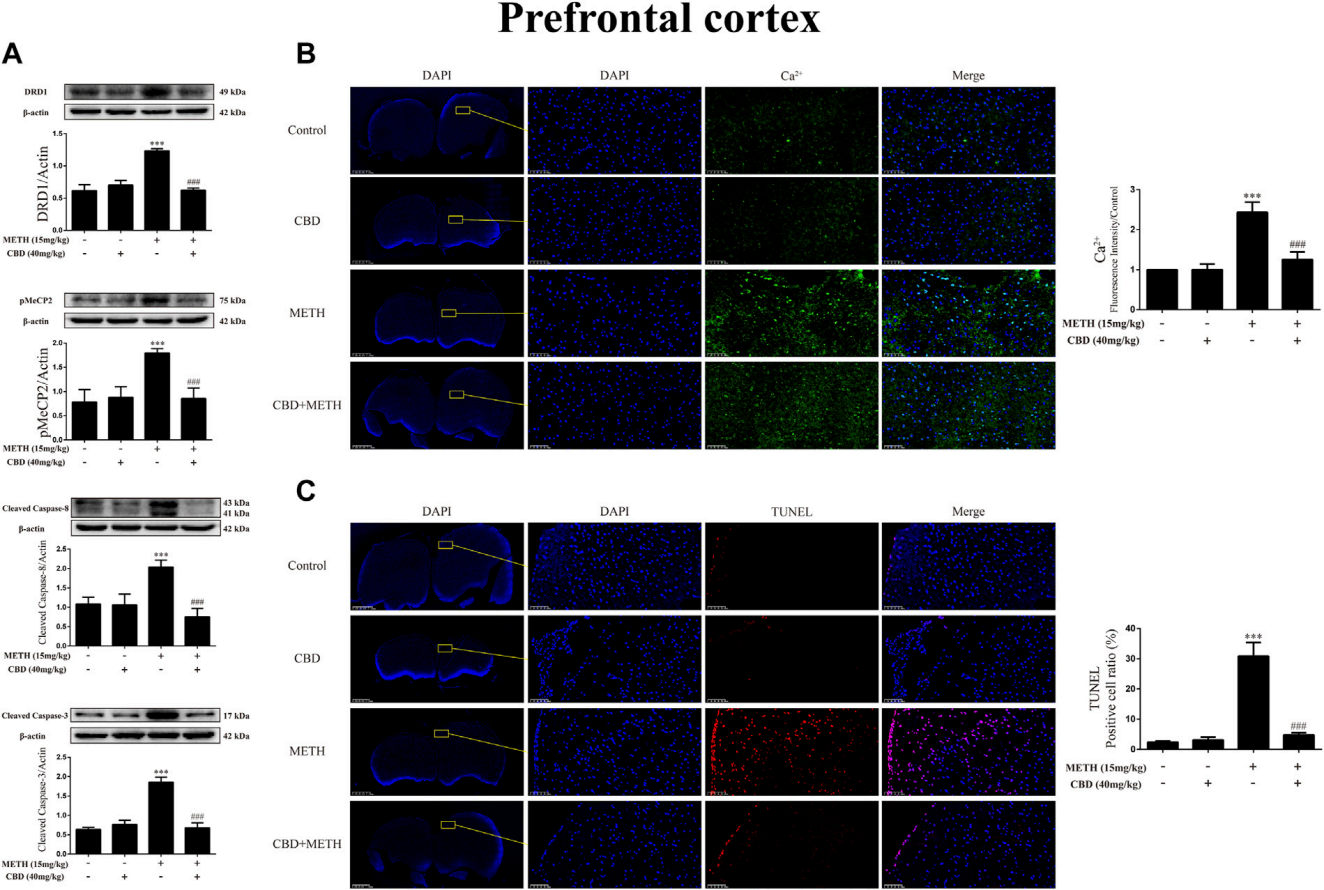
Protein, intracellular Ca^2+^, and apoptosis levels in prefrontal cortex of rats repeatedly exposed to CBD and/or METH. **(A)** Levels of DRD1, pMeCP2, and apoptosis-related proteins in prefrontal cortex. **(B)** Intracellular Ca^2+^ level in prefrontal cortex. **(C)** Apoptosis level in prefrontal cortex. ***p < 0.001 compared with control group, ^###^ *p* < 0.001 compared with METH group; Tukey HSD *post-hoc* comparison after significant ANOVA.

Repeated METH exposure affects cell proliferation and induces apoptosis in the PFC (Kim and Mandyam, 2014). Thus, to further explore the neuroprotective effects of CBD, we investigated protein expression, intracellular Ca^2+^, and apoptosis levels in the PFC of rats. Results showed that repeated METH exposure markedly increased the expression levels of DRD1 [Figure 10A; F (3, 20) = 127.723, *p <* 0.001], pMeCP2 [Figure 10A; F (3, 12) = 21.619, *p <* 0.001], cleaved caspase-8 [Figure 10A; F (3, 16) = 32.630, *p <* 0.001], and cleaved caspase-3 [Figure 10A; F (3, 20) = 173.065, *p <* 0.001] in the PFC, whereas CBD pretreatment blocked the high expression levels of DRD1 [Figure 10A; F (3, 20) = 127.723, *p <* 0.001], pMeCP2 [Figure 10A; F (3, 12) = 21.619, *p <* 0.001], cleaved caspase-8 [Figure 10A; F (3, 16) = 32.630, *p <* 0.001], and cleaved caspase-3 [Figure 10A; F (3, 20) = 173.065, *p <* 0.001]. Repeated METH exposure also increased intracellular Ca^2+^ [Figure 10B; F (3, 16) = 78.759, *p <* 0.001] and apoptosis [Figure 10C; F (3, 16) = 173.738, *p <* 0.001] in the PFC. CBD pretreatment prevented Ca^2+^ overload [Figure 10B; F (3, 16) = 78.759, *p <* 0.001] and apoptosis [Figure 10C; F (3, 16) = 173.738, *p <* 0.001] induced by METH, but had no effect when administered alone. Thus, CBD shows neuroprotective effects in the PFC of rats.

### Cannabidiol blocked DA receptors D1 expression, phosphorylation of MeCP2, Ca^2+^ overload, and apoptosis induced by methamphetamine in hip of rats

**FIGURE 11:**
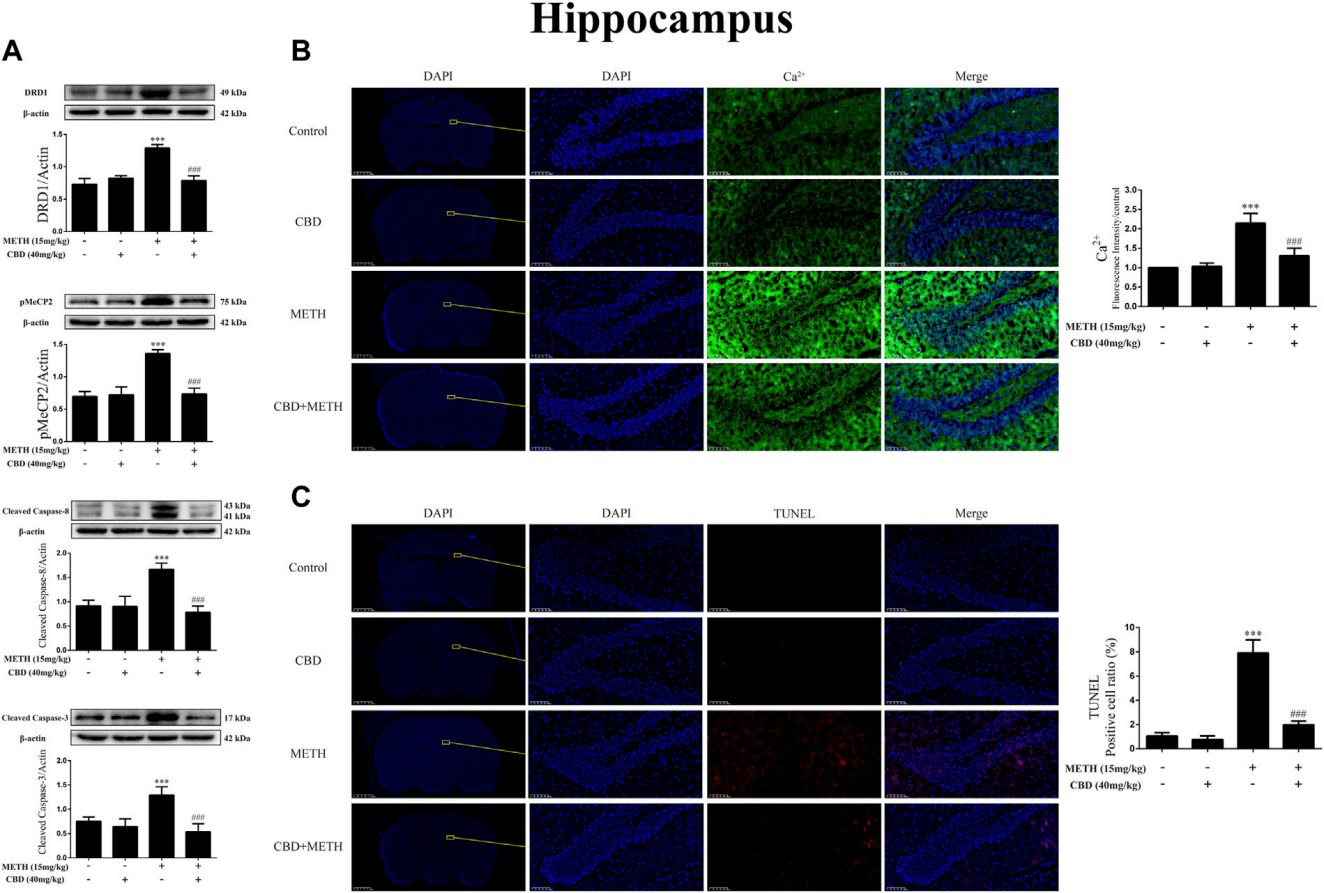
Protein, intracellular Ca^2+^, and apoptosis levels in hippocampus of rats repeatedly exposed to CBD and/or METH. **(A)** Levels of DRD1, pMeCP2, and apoptosis-related proteins in hippocampus. **(B)** Intracellular Ca^2+^ level in hippocampus. **(C)** Apoptosis level in hippocampus. ***p < 0.001 compared with control group, ^###^ *p* < 0.001 compared with METH group; Tukey HSD *post-hoc* comparison after significant ANOVA.

As CBD promotes neurogenesis in the Hip of rats under chronic METH exposure (Razavi et al., 2021) and modulates the psychoactive properties of METH *via* DA receptors in the Hip of rats (Hassanlou et al., 2021; Nouri et al., 2021), we repeated the above experiments in the Hip. Results showed that METH exposure induced DRD1 [Figure 11A; F (3, 16) = 74.578, *p <* 0.001], pMeCP2 [Figure 11A; F (3, 16) = 63.564, *p <* 0.001], cleaved caspase-8 [Figure 11A; F (3, 20) = 44.202, *p <* 0.001], and cleaved caspase-3 expression [Figure 11A; F (3, 16) = 25.338, *p <* 0.001] in the Hip, whereas CBD pretreatment blocked the high expression of DRD1 [Figure 11A; F (3, 16) = 74.578, *p <* 0.001], pMeCP2 [Figure 11A; F (3, 16) = 63.564, *p <* 0.001], cleaved caspase-8 [Figure 11A; F (3, 20) = 44.202, *p <* 0.001], and cleaved caspase-3 [Figure 11A; F (3, 16) = 25.338, *p <* 0.001]. Similarly, METH exposure also induced Ca^2+^ influx [Figure 11B; F (3, 20) = 62.846, *p <* 0.001] and apoptosis [Figure 11C; F (3, 16) = 161.004, *p <* 0.001], whereas CBD pretreatment prevented their high levels [Ca^2+^; Figure 11B; F (3, 20) = 62.846, *p <* 0.001; apoptosis; Figure 11C; F (3, 16) = 161.004, *p <* 0.001] in the Hip.

## Discussion

In the molecular docking simulations, we found that METH and CBD bound to residues of DRD1 with two overlapping binding sites. Moreover, CBD and METH competitively interacted for one of the sites. In the biological experiments, we found that METH significantly induced neurotoxicity with DRD1 activation, high MeCP2 phosphorylation at Ser421, and Ca^2+^ overload *in-vitro* and *in-vivo*. However, CBD pretreatment effectively prevented METH-induced neurotoxicity by inhibiting DRD1, pMeCP2, and Ca^2+^ influx *in-vitro* and *in-vivo*. In addition, the DRD1 antagonist SCH23390 significantly prevented METH-induced neurotoxicity, as well as pMeCP2 expression and Ca^2+^ overload *in-vitro*. In contrast, the DRD1 agonist SKF81297 strongly induced neurotoxicity, MeCP2 phosphorylation, and Ca^2+^ overload *in-vitro*, which were also blocked by CBD pretreatment.

METH is an amphetamine-type stimulant that primarily induces extracellular and cytoplasmic DA release by acting on DA transporters and vesicle monoamine transporter-2 (VMAT-2) (Kim et al., 2020). Abundant DA in the extracellular matrix and cytoplasm can produce DA quinones, superoxide anions, and hydrogen oxygen species *via* auto-oxidation, resulting in considerable oxidative damage (LaVoie and Hastings, 1999; Marshall and O'Dell, 2012). Thus, METH-induced DA release is closely related to the process of neurotoxicity (Kim et al., 2020). METH also induces the FasL-Fas death pathway and caspase-3 cleavage in rat neurons, which are mediated by DRD1 (Jayanthi et al., 2005). Here, we found that METH may bind directly with DRD1. Notably, METH exposure significantly induced apoptosis with activation of the caspase-8/caspase-3 cascade *in-vitro* and *in-vivo*, while the DRD1 antagonist SCH23390 markedly prevented the METH-induced caspase-8/caspase-3 cascade and neuronal apoptosis *in-vitro*. In contrast, the DRD1 agonist SKF81297 induced apoptosis *via* activation of the caspase-8/caspase-3 cascade *in-vitro*. These results indicate that DRD1 signaling plays a key role in METH-induced neurotoxicity, as reported in various studies (Ares-Santos et al., 2012; Ares-Santos et al., 2013; Friend and Keefe, 2013; Nguyen et al., 2018). However, some evidence suggests that other molecular pathways may also contribute to cell death under METH-induced neurotoxicity, including oxidative stress, mitochondrial stress, ER stress, UPS dysfunction, transcription factor changes, and autophagy (Jayanthi et al., 2021). Here, the DRD1 antagonist SCH23390 did not completely block the effects of METH on Ca^2+^ influx, MeCP2 phosphorylation, caspase cascade, or neuronal apoptosis. Interestingly, DA induces the expression of DRD1 at low concentration (Sidhu et al., 1999) and functional activity of DRD1 also determines DA release (Shen et al., 2022). This positive feedback mechanism may partially explain why the DRD1 antagonist SCH23390 and DRD1 agonist SKF81297 affected DRD1 expression in our study.

On the other hand, growing evidence indicates that MeCP2 contributes to METH-induced behavioral disorders in rodents (Lewis et al., 2016; Wu et al., 2016; Fan et al., 2020). We observed serious stereotyped behavior in rats under repeated METH exposure, consistent with previous research (Roberts et al., 2010; Qiao et al., 2014). Notably, METH strongly induced stereotyped behavior in rats, with high phosphorylation of MeCP2 at Ser421 in the PFC and Hip. The DRD1 antagonist SCH23390 significantly blocked METH-induced phosphorylation of MeCP2 at Ser421 in the primary neurons of rats. In contrast, the DRD1 agonist SKF81297 strongly induced MeCP2 phosphorylation at Ser421 *in-vitro*. These results suggest that the phosphorylation of MeCP2 at Ser421 is mediated by DRD1, consistent with previous research (Deng et al., 2010). As the phosphorylation of MeCP2 at Ser421 is calcium-dependent (Chen et al., 2003; Buchthal et al., 2012), we further investigated the level of intracellular Ca^2+^, and found that METH exposure strongly increased Ca^2+^ influx *in-vitro* and *in-vivo*. However, DRD1 inhibition significantly reduced the high level of intracellular Ca^2+^ caused by METH, while DRD1 activation markedly increased intracellular Ca^2+^ in the neurons. These results imply that DRD1-mediated phosphorylation of MeCP2 at Ser421 involves calcium signaling. Several studies suggest that changes in IEGs may also contribute to METH-induced neurotoxicity (Deng et al., 1999; Jayanthi et al., 2005; Jayanthi et al., 2021). Interestingly, research has shown that phosphorylation of MeCP2 at Ser421 mediates the transcriptional activation of IEGs in psychostimulant-injected mice (Deng et al., 2010). Thus, these findings reveal the potential mechanism underlying METH-induced neurotoxicity involving DRD1-mediated calcium-dependent phosphorylation of MeCP2.

CBD exhibits excellent therapeutic potential in neuropsychiatric disorders such as epilepsy, PD, AD, depression, anxiety, psychosis, and drug dependence (Elsaid et al., 2019; Premoli et al., 2019; Vitale et al., 2021). Under METH exposure, CBD shows neuroprotective (Razavi et al., 2021), antioxidative, and anti-inflammatory properties (Majdi et al., 2019; Karimi-Haghighi et al., 2020). In addition, CBD can restore the adaptation to threshold doses caused by repeated METH exposure (Khanegheini et al., 2021). Our results showed that CBD pretreatment prevented METH-induced caspase-8/caspase-3 cascade and apoptosis *in-vitro* and *in-vivo*. During abstinence periods, CBD has been shown to promote neurogenesis in the Hip dentate gyrus in rats under chronic METH exposure (Razavi et al., 2021). In the current study, we found that CBD pretreatment markedly prevented the increase in caspase-8/caspase-3 cascade and apoptosis in the Hip of rats induced by repeated METH exposure. Given our results and those of the abovementioned studies, CBD shows substantial neuroprotective effects on METH-induced neurotoxicity. However, one major obstacle regarding CBD research is our relatively poor understanding of the mechanisms underlying its therapeutic potential. As CBD has a low-binding affinity toward traditional cannabinoid receptors (CB1-R and CB2-R) (Izzo et al., 2009; Bian et al., 2019; Vitale et al., 2021), determination of the main active target(s) of CBD is essential. Evidence indicates that CBD modulates DA receptors (Hassanlou et al., 2021; Nouri et al., 2021), Sigma-1 receptors (Yang et al., 2020), and TLR4 (Majdi et al., 2019). Here, we found that CBD may directly bind with DRD1 and compete with METH for the PHE-313 binding site. These two partially overlapping binding pockets likely affected ligand and receptor binding. This may explain why the type of interactions between CBD and DRD1 residues were variable when CBD and METH docked with the DRD1 model. Previous molecular docking evidence suggests that CBD is a potential binding ligand for DRD3 (Bian et al., 2019). Therefore, CBD may exert broader effects on DA receptors. In addition, our biological experiments showed that CBD also exhibited significant preventive effects on SKF81297-induced Ca^2+^ influx, MeCP2 Ser421 phosphorylation, caspase-8/caspase-3 cascade, and apoptosis. These results suggest that the preventive effects of CBD on neurotoxicity may be mediated by DRD1.

However, METH-induced neurotoxicity not only involves direct damage to neurons, but also the participation of neuroinflammation (Shaerzadeh et al., 2018; Kim et al., 2020). Previous studies have shown that METH induces neuroinflammation *via* TLR4-or Sigma-1 receptor-related pathways (Kim et al., 2020). In addition, research suggests that METH-stimulative neuroinflammation may involve the participation of D1-like DA receptors (Wang et al., 2019), which are highly expressed in the microglia (Huck et al., 2015). Thus, the contribution of DRD1 to METH-induced neurotoxicity may not be limited to neurons. Furthermore, the phosphorylation of MeCP2 at Ser421 is also observed in microglia after psychostimulant exposure (Cotto et al., 2018). Therefore, the regulatory effects of DRD1-mediated phosphorylation of MeCP2 on METH-induced neurotoxicity may not be limited to neurons but could exert more global effects on multiple types of cells, providing a clear direction for future research.

In conclusion, our results suggest that METH induces neurotoxicity *via* DRD1-mediated calcium-dependent phosphorylation of MeCP2 at Ser421. Moreover, CBD significantly prevents METH-induced neurotoxicity *via* modulation of DRD1. Future research should focus on the contribution of neuroinflammation to neurotoxicity and explore the anti-inflammatory effects of CBD under METH exposure.

## Data Availability

The original contributions presented in the study are included in the article/supplementary material, further inquiries can be directed to the corresponding authors.
